# On the Connexion Between Morbid Physical and Religious Phenomena

**Published:** 1855-01-01

**Authors:** J. F. Denham


### ON THE CONNEXION BETWEEN MORBID PHYSICAL AND RELIGIOUS PHENOMENA.

### No. II. of a Series.

BY THE REV. J. F. DENHAM, M.A., F.R.S., &c.

In the last paper on this subject it was attempted to show this connexion in regard of those religious phenomena which are of a gloomy or distressing nature: it will now be endeavoured to trace it in reference to those which are of a more pleasing and elated kind. The distinction of both these classes of religious phenomena is, that they exist separately from any definite perceptions of the understanding, and consist wholly of unintelligent feelings and emotions. It is thus stated by two eminent divines of the Church of England:—"If you desire to know the differences between the heaviness of a melancholy humour and affliction of conscience for sin, take notice of such as these:—the melancholy man is extremely sad, and *knows not why*. He is full of fear, doubts, distrust, and heaviness, *without any true or just ground*, arising only from the darkness and disorder of the imagination—the grisly fumes of that black humour in the brain. But a broken heart, in almost every case, can readily tell you the particular sins that made it bleed. His trouble is ever upon cause clear and evident. A melancholy man will ride many miles, walk many hours, and at length be able to give you no account of the exercise and discourse of his mind, or what his thoughts have been all the while."* Archbishop Sharp thus states the same distinction in regard of elated emotions:—"How shall we be able to know when the joy and satisfaction we feel in the exercise of religion doth arise from the Holy Spirit, and when from our own tempers? This is a material question, and thus I answer it. All those joys that we *can give no good account of*, that arise in our minds we know not how or wherefore; and likewise all those joys which do not more incline us to love God and our neighbour, do not dispose us to hate vice and impurity, and especially the more spiritual impurities of pride and self-love, which we may labour under;—I say, all these joys and consolations, how high and rapturous soever they be, are justly to be suspected by us, as the pure results of our own heated temper, the ebullition of our own animal spirits. The peace and joy of the Holy Ghost is *always rational*, there is some good ground, some solid foundation for it in the mind of the man that feels it; which foundation is a good conscience, a being able to satisfy ourselves, from the testimony of our hearts and lives, that we are sincere and unfeigned in our desires and endeavours to approve ourselves to God as his faithful servants. It is never a barren, ineffectual joy, a joy that only amuses and pleases us without making us better; and if that joy which we sometimes feel in the exercise of devotion be not of this nature, and have not these qualities, let it be otherwise never so affecting and transporting, we cannot be assured that it is from God; nay, rather we have reason to conclude that it is the natural effect of our own temper and natural powers. It is past dispute that these overflowing joys and comforts that are sometimes felt upon the application of our minds to spiritual things, are the mere effects of a heated brain and a raised imagination, for it is certain that in some persons these effects, even in the highest degree of them, owe their production to no higher a cause. Witness the transports of joy and the pleasures—even to ecstasy—which many enthusiastical persons have felt, or at least have given out that they have, in the exercise of the grossest superstition (of a false religion),

* Bolton's Treatise on Affliction of Conscience. Written 1620. Edited, with Introduction and Memoir of the Author, by Rev. J. F. Denham. London. 1831. page 142, &c.

and which many of the same complexion have experienced likewise in the true religion; who yet have been persons of none of the best morals, but in truth wholly devoid of the spiritual life. Now, I say, to attribute these raptures and ecstasies of joy in such persons to the Holy Spirit of God, will be very hard, at least now-a-days, when miraculous powers have ceased. No; certainly, all spiritual joy is not the joy of the Holy Ghost; a man may be sometimes so full of joy that his soul is even ready to break its prison, and yet, for all that, be not a whit the more acted by a divine spirit."*

This characteristic, then, of morbid religious feelings, both melancholy and elated, that neither of them are rational, being assumed, it seems to follow as a just conclusion that both kinds of them are symptomatic of disease; and this conclusion in regard of the latter kind of them seems strengthened by the following considerations; they are always accompanied by similar morbid feelings and emotions in regard of other objects and subjects, and with unaccountable sympathies and antipathies; are chiefly incidental to persons of weak or uncultivated minds, of a highly excitable temperament naturally, or in consequence of debilitation, to the subjects of constitutional indolence aggravated by the absence of regular and active employment; to females and to effeminate males of sedentary avocations; to miners and others living and labouring in a vitiated atmosphere, and amid cheerless or unvarying scenes. Such feelings are often combined with hysteric, &c., affections, and with evident indications of functional or organic diseases, especially of the organs of circulation, and of the arterial system connected with the stomach and its region. They are frequently found along with a debility of moral principle in regard of truthfulness, honesty, temperance, or with an insensibility to relative duties. Such persons have generally imbibed the notion that their peculiar feelings and emotions are the infallible index of piety and indubitable marks of divine favour. To use the words of an eminent prelate,—"They seem to think themselves bound to feel all they read in the Scriptures, without regard to the difference between the present and past economy of things;"† and possess, for a time at least, the power of raising in themselves these ecstatic emotions by an effort of their own will. They often exhibit a tendency to *malingery*, and in other respects possess the simulative sensibilities of actors and actresses in remarkable perfection. I am emboldened by theological and medical authorities to express my conviction that the generality of those persons in whom what are called the religious emotions are peculiarly developed, are the subjects of physical disease; and that an immense number of religious books, sermons, &c., are indebted for their popularity to the diseased susceptibilities of their readers and hearers. Such morbid states are often peculiarly manifested in regard of prayer and psalmody, the former being in many cases a merely self-mesmerizing process of unintelligent emotions, and the latter a mere physical hilarity irrespective of words and ideas. There is not a town, or perhaps a village, in the empire in which such instances of morbid religious phenomena are not to be found; and it is impossible to set a limit to the delusions which the subjects of them may undergo. The great evil of them, next to their fallacy, consists in their tendency to destroy the true religious capacity, and in their powerful conduciveness, in common with all other kinds of excitement, to those vices and crimes to which the temperament, whether of mind or body, may be liable, or to which the circumstances of individuals may peculiarly expose them: nor should their too-possible termination in insanity or suicide be forgotten.

The difficulty, if not impossibility, of effectually ministering to confirmed cases of this nature, arises from the fixed belief by the patient (with which they are commonly attended) of the sacred origin of such feelings, and from the

* Casuistical Sermon, III., vol. 3. London. 1716.
† Bishop Warburton on Grace, page 112.

gratification which they so readily and largely afford to his perverted mind. Our prospect of success is limited to the incipient stages of his disease, when, perhaps, an occasional scepticism intrudes itself respecting the supernatural origin of his emotions. The proximate cause of the malady would seem to be what has been called "reflective consciousness, or internal observation." Positive consciousness, as in the case of *mauvaise honte,* disturbs and perverts the operation of all our mental and moral powers, as also of our physical. Introspection produces that emotion to which the state of the physical constitution is at the time inclined, especially if the desirableness of that emotion be also previously believed. When this examination of the consciousness is regarded as a duty, in consequence, perhaps, of a misapprehension of certain passages of Scripture, a morbid state of the feelings is promoted with every act of it. The means of cure or prevention, as indicated by the nature of the disease, are to substitute religious acts and moral duties, as these are dictated by reason, conscience, and the Scriptures, for all attention to the phenomena of consciousness, reveries, or abstract contemplations. The perusal of religious publications of a sentimental or romantic character should be strictly prohibited, as well as all attendance upon those services, sermons, orations, &c., which appeal rather to the imagination than to the judgment and reason. Change of scene and cheerful society, scientific lectures and active occupation, both of the mind and body, upon external objects, seem fittedto divert the mind's attention from itself, and to break the chain of its diseased associations. Nor is the use of sarcasm utterly unavailing, especially that kind of it generally too applicable to such cases, which is derived from the inferior moral conduct of those who make pretensions to the most spiritual emotions. But no applications to the mere mental constitution of the patient will be effectual unless aided by an alleviation of that physical malady which is the real root of the disorder. This can only be treated according to its particular nature. As a general rule, however, the entire abstinence from all physical stimulants, to some of which such patients often show a marked propensity, may be safely recommended. All direct tonics, as well as all opiates, generally augment the disease. Tea is frequently injurious. The late eminent clergyman, Mr. Cecil, speaks of "females sitting over a fire all day, and drinking tea, and then mistaking their morbid feelings for divine influences."*

I beg to add the application of the foregoing principles, &c., to the subject of life's closing scene, in which a large portion of the community expect that the religious sincerity of the sufferer will become apparent, the power of religion will, by divine favour, be peculiarly evinced, and an antepast will be afforded of future bliss, which will even leave its last gleam upon the very countenance of the departed. This expectation is nourished by obituaries and memoirs, and in direct opposition to numerous well-known instances of persons of undoubted worth and piety, in regard of whose last feelings and post-mortem aspect "the King of Terrors" plainly showed that he is no respecter of persons. I will merely advert to some circumstances and considerations which may lead to a distrust of all the appearances of the death-bed, and of all inferences from them. These considerations are partly derived from the nature of the disease and the correspondent alterations it produces of the blood, &c., and consequently of the action of the brain, and thereby of the mental feelings and perceptions, whether as hyper-oxygenated or super-carbonized: of which former state every regular case of phthisis of the lungs affords an illustration, as does also every case of obstructed liver, of the latter. I have rarely seen the former case, and its characteristic exhilaration of spirits and exaltation of ideas, even to the last moment of life, without being reminded of the remark I once heard made by an eminent medical attendant, that "consumption is a poetical

* Cecil's Remains.

disease;" nor of the latter, without remembering the effects on the mind attributed to *black-bile* by the ancients. To these causes of cerebral and mental disturbance must be added the solitude and sameness of the sick-room and its sickly atmosphere, the medicines administered, the peculiar food prescribed, the stimulants ordered and often administered in additional quantities by friends and attendants, and the natural tendency of debility itself to produce delirium; and we have only need to consider the inevitable consequences on the mind, perhaps of a long course of such treatment, in order to feel convinced that but little reliance is to be placed upon the dying man's expressions or feelings, either as being, when "triumphant," a prelude to the bliss of Paradise; or as being, when despondent, the dark indication of "a monstrous life."

This reliance becomes still further diminished by the well-known quality of strongly-excited feelings to represent themselves to the mind as ideas, and even as actual impressions on the senses, and of any violent alternations of the feelings to assume the form of a dialogue to the imagination. I subjoin a few published and well-attested incidents illustrative of the foregoing observations, &c. The first is of the effects of delirium.

"Samuel Hitchens, a smith by trade, was taken ill, and caught a *malignant fever, in which* he cries out, 'I have not the least doubt of my salvation.' He cries out aloud, 'Open the heaven, O my God, and come down into my soul. Come, Father, Son, and Holy Ghost, and *plunge me into God!*'"

The following seems an instance of false perception:—

"A man fell ill of a high fever; addressing himself to the people around him, he says, 'Can't you see Jesus Christ coming, with an innumerable company of angels, and the golden banner displayed? They are coming to carry me to the bosom of my God. Open their eyes, O God, that they may see them. I am whiter than snow,—I am washed in the blood of my Redeemer. Why, I am all God."—"Bishop Lavingdon's Enthusiasm," &c., Part III., p. 93.

I subjoin the following valuable remarks upon the feelings in death:—

"I would not alter my opinion of a man's spiritual state, whom I had thoroughly known before, for the manner of his death. The end of a holy life and unblamable conversation may not appear in the eye of man so calm and comfortable as was expected. Some such men may end their days in ravings, impatiencies, and other strange behaviours. The fiery distempers of their hot disease may sometimes, even in the saints of God, produce furious conduct, fearful distractions, and despairful speeches, these being the natural effects and issues of melancholy excesses, frenzies, and burning fevers."* The following observation of Lord Bacon may confirm the view already taken of the delusibility of human feelings, and induce us to rely on the more solid ground of conduct and duty:—

"'The mind, darkened by its covering, the body, is far from being a flat, equal, and clear mirror, that receives and reflects the rays without mixture, but rather a magical glass, full of superstitions and apparitions.'"†

* Bolton, p. 152, &c. † Works, vol. I., p. 132. Shaw's edition.

